# Supplementary material for: Reconfigurable MEMS Fano metasurfaces with multiple-input–output states for logic operations at terahertz frequencies
Source: Nat Commun. 2018 Oct 3;9:4056. doi: 10.1038/s41467-018-06360-5 (PMC6170453; doi:10.1038/s41467-018-06360-5)
Supplement: Supplementary file 1 — Supplementary Information [file 41467_2018_6360_MOESM1_ESM.pdf]

Supplementary Information

Reconfigurable MEMS Fano metasurfaces with multiple-input-output states for logic operations at terahertz frequencies

Manukumara Manjappa[1,2], Prakash Pitchappa[1,2], Navab Singh[3], Nan Wang[3], Nikolay I Zheludev[2,4], Chengkuo Lee[5,6], Ranjan Singh[1,2,*]

[1]*Division of Physics and Applied Physics, School of Physical and Mathematical Sciences, Nanyang Technological University, 21 Nanyang Link, Singapore 637371, Singapore.*

[2]*Centre for Disruptive Photonic Technologies, The Photonics Institute, 50 Nanyang Avenue, Nanyang Technological University, Singapore 639798.*

[3]*Institute of Microelectronics, 11 Science Park Road, 117685, Singapore.*

[4]*Optoelectronics Research Centre and Centre for Photonic Metamaterials, University of Southampton, Highfield, Southampton SO17 1BJ, United Kingdom.*

[5]*Department of Electrical & Computer Engineering, National University of Singapore, 4 Engineering Drive 3, 117576, Singapore.*

[6]*Center for Intelligent Sensors and MEMS (CISM), National University of Singapore, E6 #05-11F, 5 Engineering Drive 1, Singapore 117608, Singapore*

* Corresponding author: *ranjans@ntu.edu.sg.*

Supplementary Figure 1.

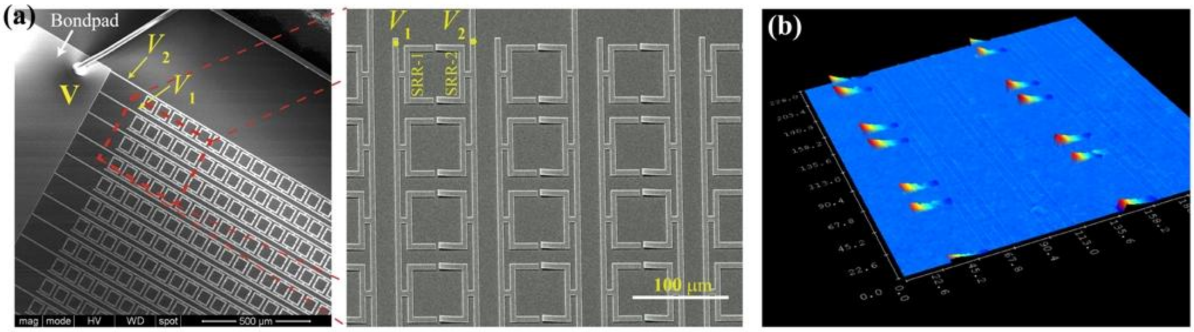

(a) Scanning electron microscope (SEM) and (b) 3D profilometer images of the fabricated microelectromechanical system (MEMS) Fano-metamaterial depicting the maximum asymmetric state of the system. One of the cantilevers (say, SRR-1) is snapped down by applying voltage V_1, whereas the adjacent cantilever (SRR-2) is in the released state with $V_2 = 0$.

Supplementary Figure 2.

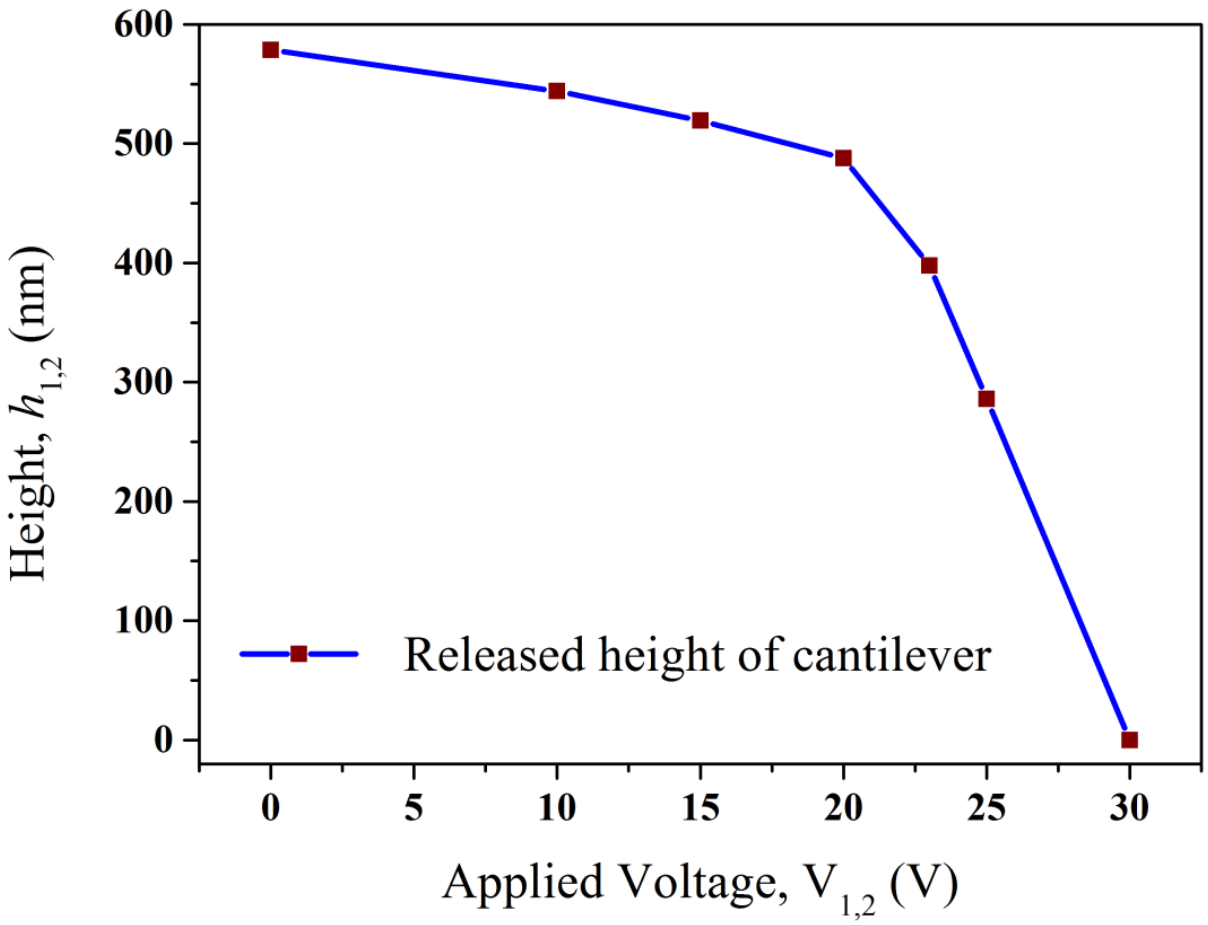

Experimentally measured released heights of the cantilever for the various values of the applied voltages. The graph shows the nonlinear change in the height of the released cantilever with respect to the applied voltage. When the voltage is applied on the bimorph cantilevers, cantilevers experience competing electrostatic attraction force and the restoring force on the bimorph structure as described in the methods section of the article. This dictates the observed nonlinear actuation response of the cantilevers with respect to the applied voltage.

Supplementary Figure 3.

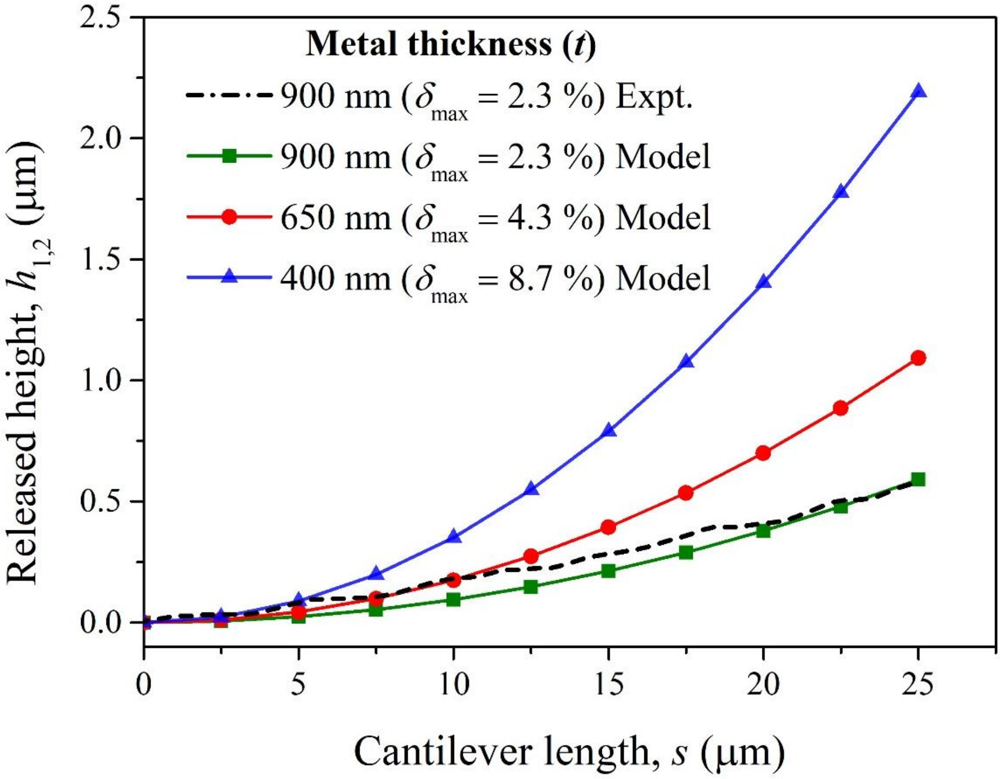

Theoretically modelled[1] and experimentally measured released height profile for the bimorph cantilevers of different metal thicknesses. The bimorph structure bends up forming a curvature that widens with decreasing thickness of the metal. The dash black line represents the experimentally measured released height profile of the cantilever of thickness 900 nm measured using the Lyncee Tec. reflection digital holographic microscope (R-DHM). The theoretical model[1] finds the good agreement with the measured data.

Supplementary Figure 4.

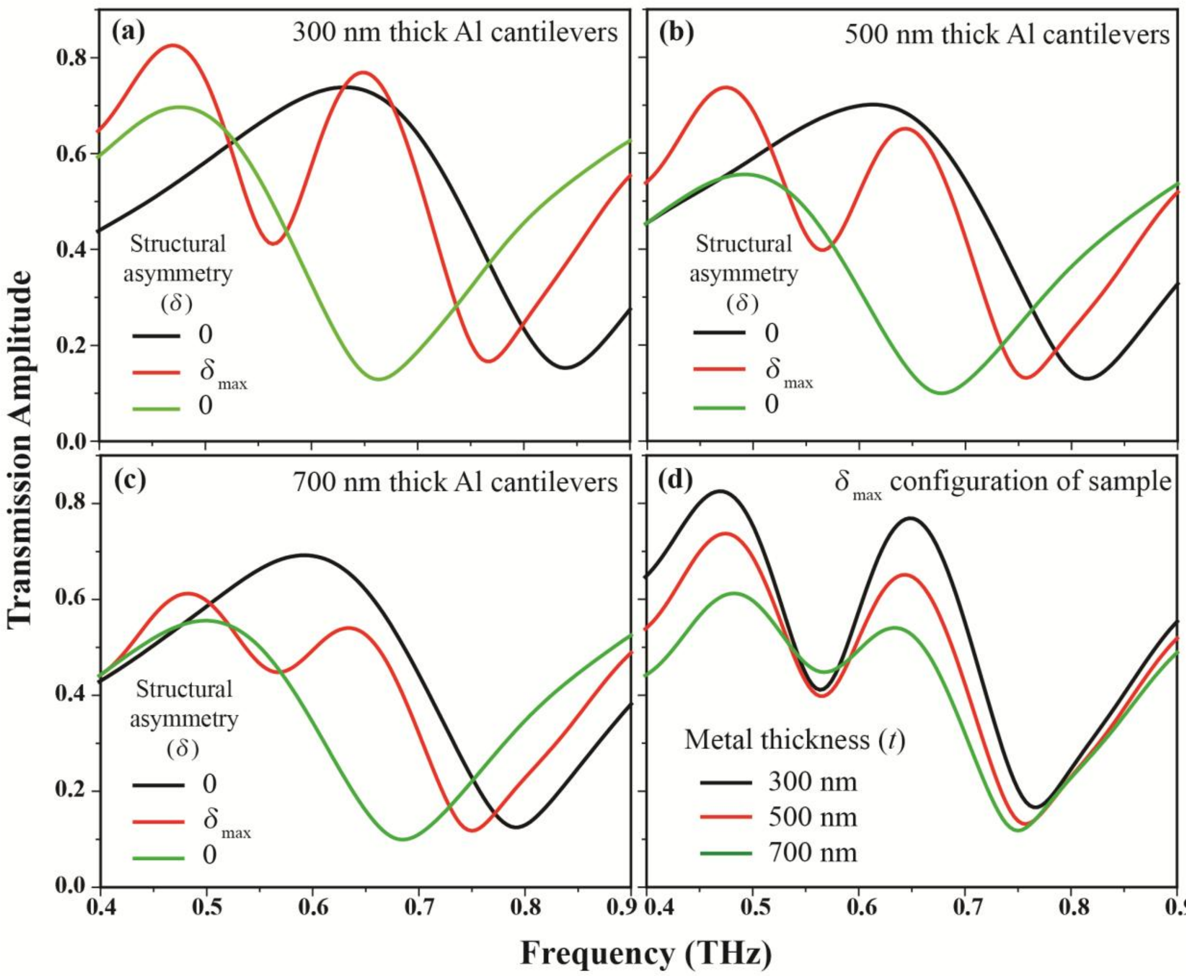

Experimentally measured THz transmission data for various metal thicknesses of cantilevers in MEMS Fano-metamaterial: (a)-(c) Depicts the experimentally measured transmission spectra for different thicknesses (300, 500 and 700 nm) of aluminium (Al) SRR cantilevers (SRR-1 and SRR-2) in both released states ($\delta = 0$), one released-one snapped state ($\delta = \delta_{max}$) and both in the snapped states ($\delta = 0$), respectively. (d) Shows the measured transmission spectra for various thicknesses of Al cantilevers (300, 500 and 700 nm) at the maximum asymmetry state ($\delta = \delta_{max}$) of the sample. 300 nm thick metal sample exhibits stronger Fano resonance amplitude as it exhibits maximum released height and hence maximum asymmetry.

The electro-optical response from all these samples shows the similar behaviour in terms of resonant characteristics, except the gradual red-shift and different strength of Fano resonance features. For 300 nm thick metal sample, the release height will be greater than the metal thicknesses of 500 and 700 nm samples and hence it exhibits maximum asymmetry and maximum strength in the amplitude of Fano resonance, as shown in Supplementary Figure Fig. 4 (d). The red shift seen in the resonances of the samples for increasing metal thicknesses is due to increase in the capacitance for the thicker metal sample possessing smaller released heights.

Supplementary Figure 5.

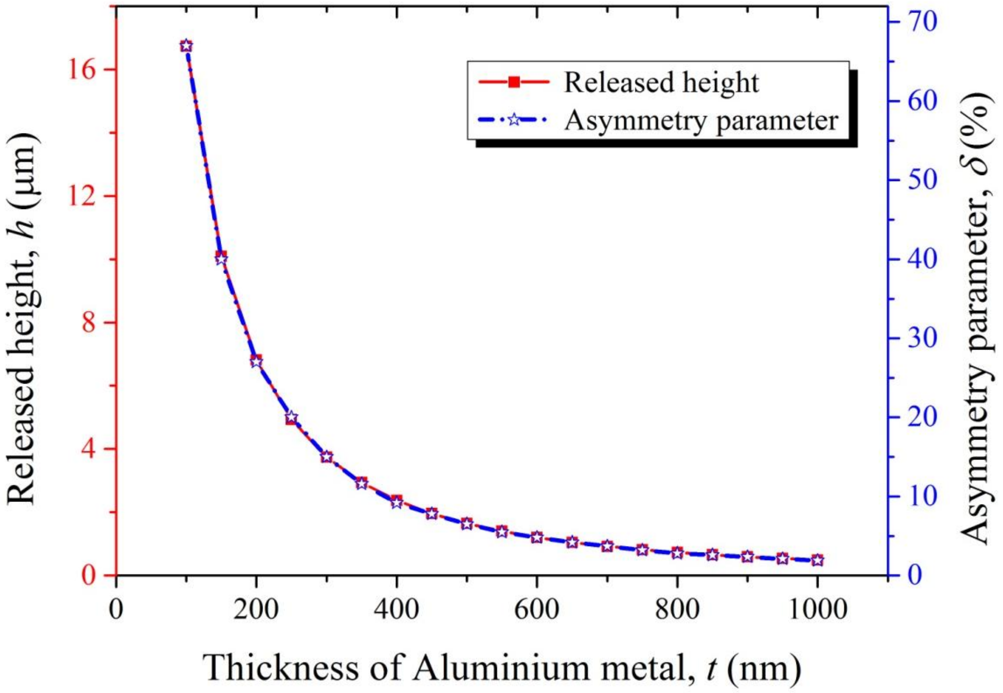

The figure represents the extent of release heights (red solid squares) for varying thicknesses of the metal arms calculated using the theoretical model[1], while keeping all other geometrical parameter constant. The plot predicts that thinner the metal lines results in larger released heights of the cantilevers. Thus, the maximum asymmetry parameter in the

proposed design can be tuned by choosing the desired metal thickness of the cantilever, as shown by the curve with open blue asterisks. The overlapping of the curves indicates the asymmetry parameter is proportional to the released heights. The lowest thickness of the cantilever in the current design is limited by the skin depth of the metal used, which in our case is aluminium metal that possesses skin depth of 82 nm at 1 THz.

Supplementary Figure 6.

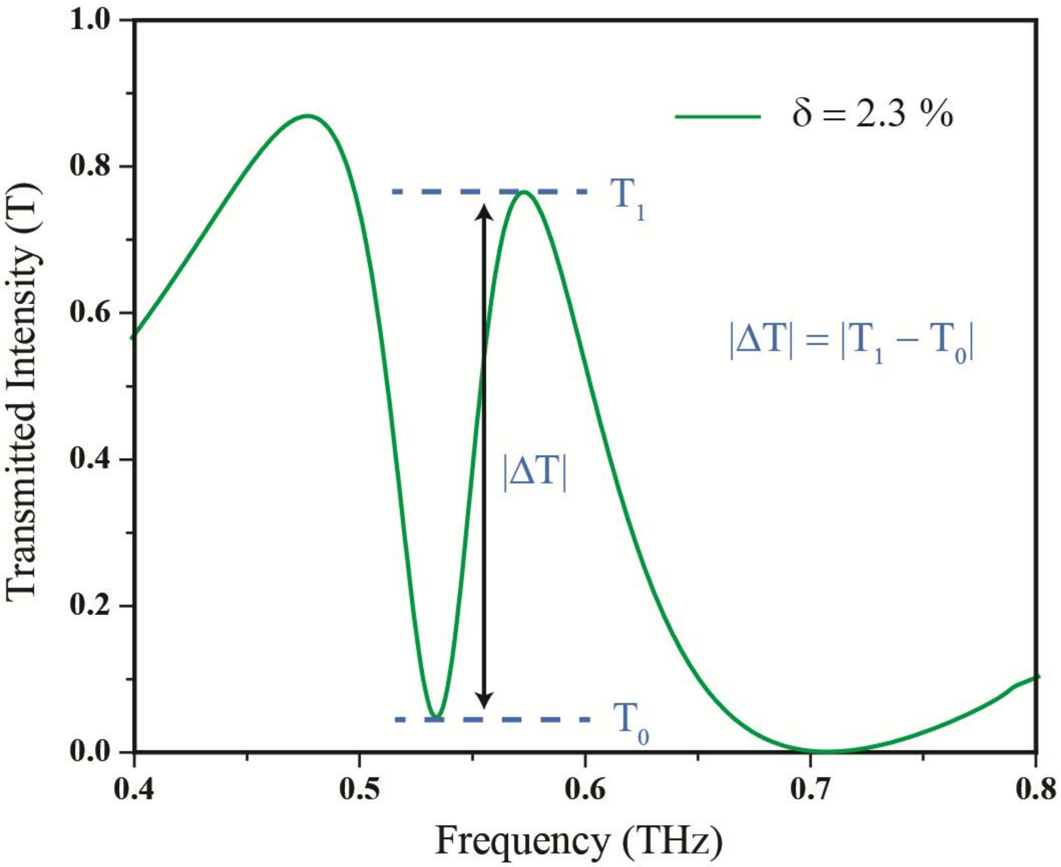

Calculation of peak to peak transmission intensity (transmittance) of the Fano resonance. The Fano peak to peak intensity $|\Delta T|$ is determined by taking the difference between the intensities T_1 and T_0 as shown in this figure. Here, transmitted intensity spectrum is shown for the maximum asymmetry ($\delta = 2.3$ %), where the amplitude/intensity of the Fano resonance is the maximum.

Supplementary Figure 7.

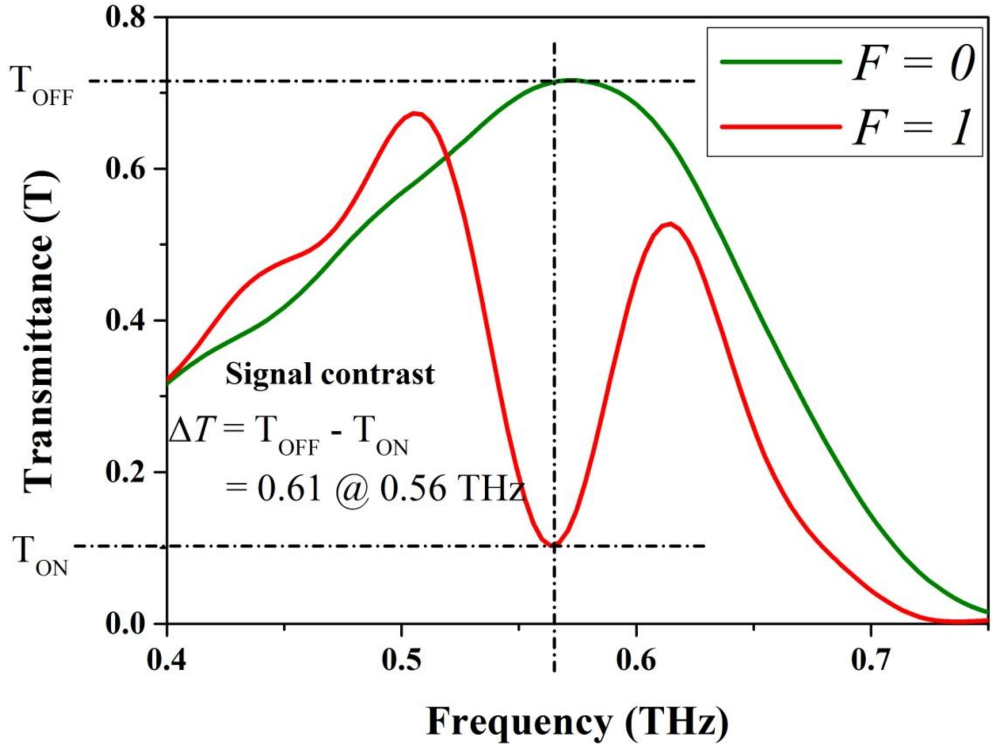

Experimentally measured intensity contrast for the *F = 0* and *F = 1* logic output states: Instead of the terahertz time domain (THz-TDS) source (used in our measurements, that measure the THz-time pulse), a single frequency source (continuous THz source) can be employed as the read-out THz beam and the output power can measured from the photo-detector, which can show a good contrast of change in transmittance, $\Delta T = 0.61$ ($T_{OFF} = 0.71$ for $F = 0$ state and $T_{ON} = 0.1$ for $F = 1$ state, normalized to input intensity of T = 1) between the OFF ($F = 0$) and ON ($F = 1$) states at 0.56 THz.

Supplementary Figure 8.

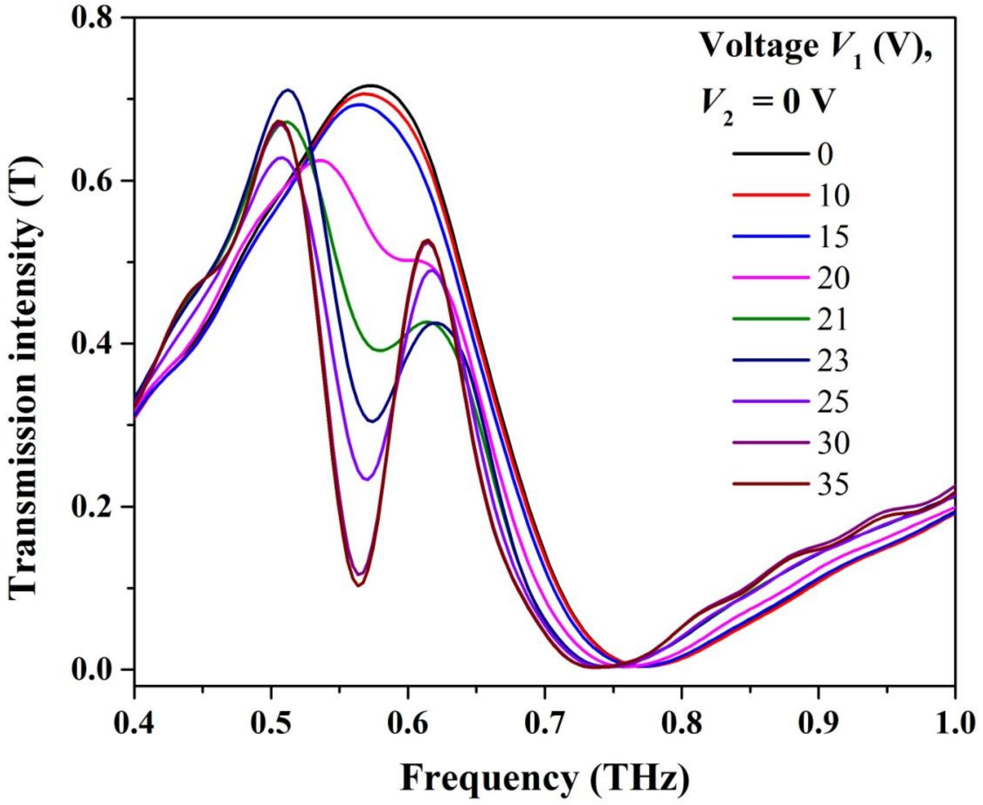

Experimentally measured transmission intensity (transmittance) spectra for increasing asymmetry (δ) configuration. The measured transmitted intensity spectrum used to plot the multiple-input-output curves given in the Figure 3(a) of main article, for increasing pathway of differential voltage (ΔV). For the increasing δ configuration, the voltage V_1 that dictates the structural state of SRR-1 is varied by keeping the voltage V_2 of SRR-2 fixed at 0 V.

Supplementary Figure 9.

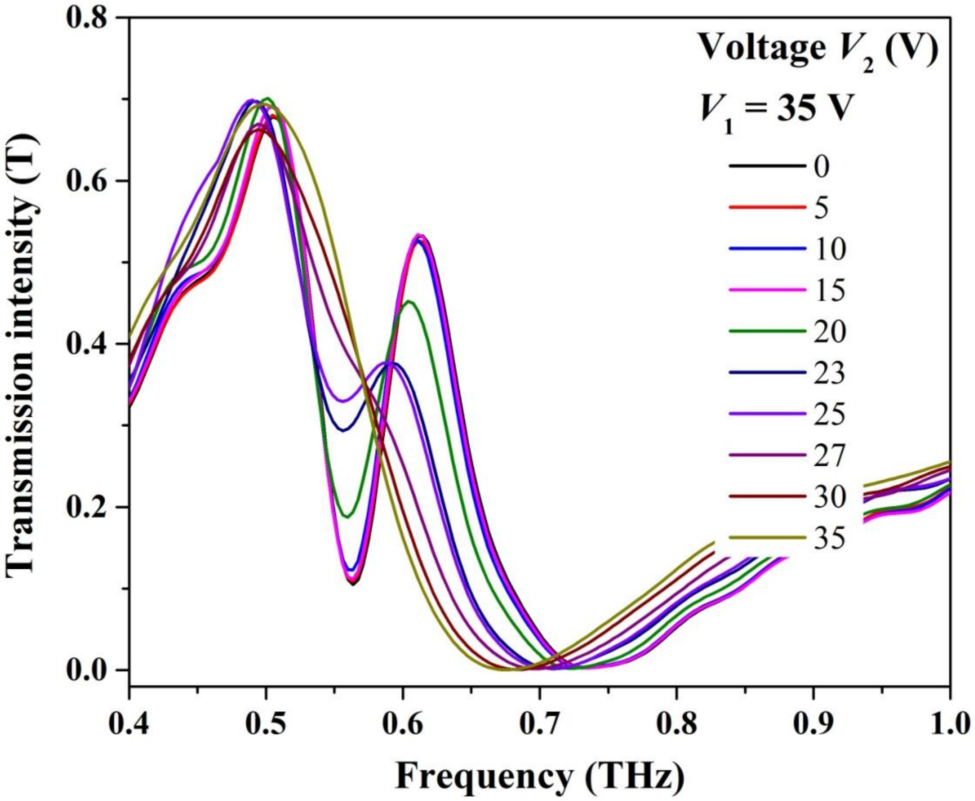

Experimentally measured transmission intensity (transmittance) spectra for decreasing asymmetry: The measured transmitted intensity spectrum used to plot the multiple-input-output curves given in the Figure 3(a) of main article for the decreasing pathway of differential voltage (ΔV). For the decreasing pathway configuration, the voltage V_1 that dictates the structural state of SRR-1 is fixed at the maximum value (35 V) and voltage V_2 of SRR-2 is varied from 0V to 35 V.

Supplementary Figure 10.

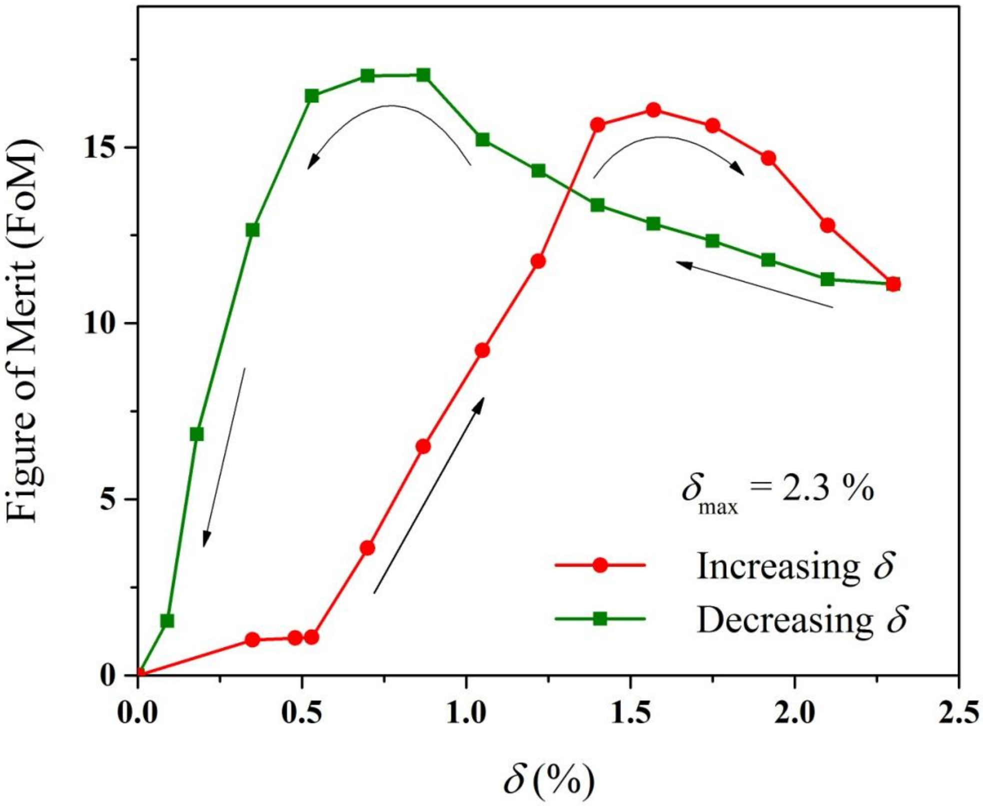

MIO behaviour in FoM. The figure depicts the variation in the figure of merit (FoM) values of the Fano resonance with respect to the increasing and decreasing pathways of δ. The FoM[2] is defined as the product of the Q-factor and the peak to peak intensity (ΔT) of the Fano resonance that represents the optimized strength of the Fano resonance for sensing and nonlinear applications.

Supplementary Figure 11.

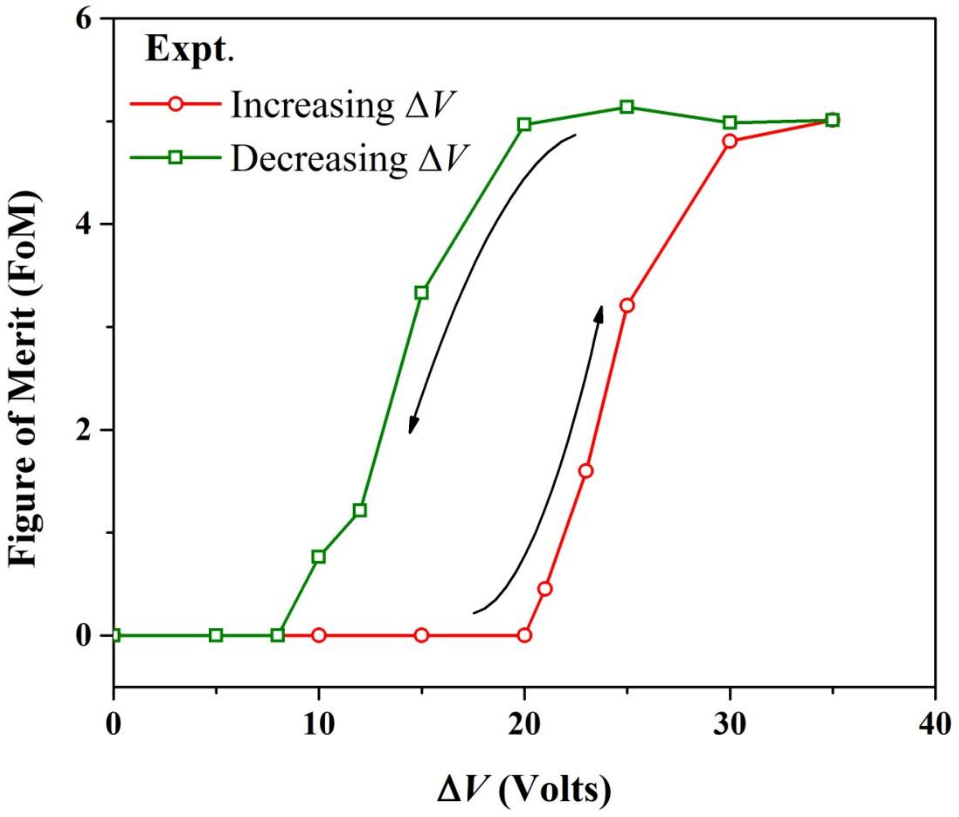

Experimental data of MIO behaviour in FoM. The figure depicts the variation in the experimentally calculated figure of merit (FoM) values of the Fano resonance with respect to the increasing and decreasing pathways of differential voltage $\Delta V = |V_1\text{-}V_2|$.

Supplementary Figure 12.

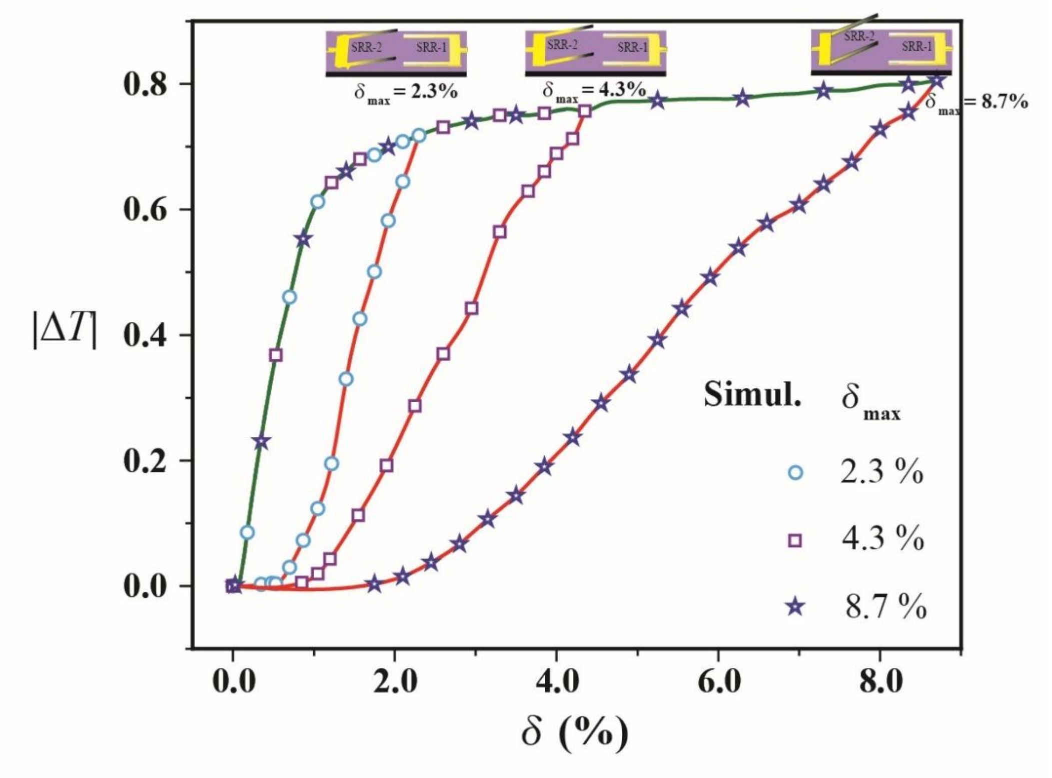

Tuneable MIO characteristics in the far-field intensity of Fano resonance: The ability of accessing different release heights by varying the thicknesses and the voltage state of the cantilever gives a great advantage in precisely tuning the area/width of the MIO curves observed in the optical properties of the Fano resonance. The figure shows the various pathways of numerically extracted far-field Fano peak to peak intensity variation with respect to the structural asymmetry parameter (δ) of the metamaterial. For increasing values of maximum asymmetry state (δ_{max}), area/width of the MIO loop can be tuned adequately. The red lines and green line represent increasing and decreasing pathways for the asymmetry parameter, respectively.

Supplementary Figure 13.

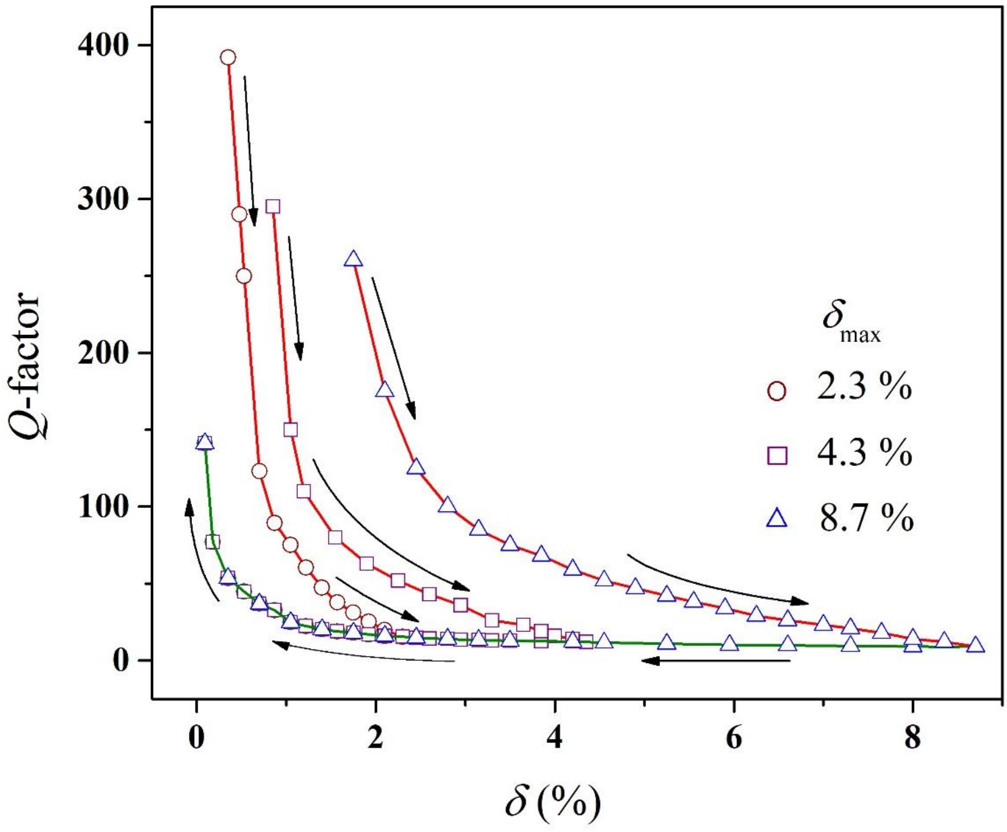

Tuneable MIO characteristics in *Q*-factors. The figure shows the various pathways of *Q*-factor variation calculated from the simulated Fano resonance spectrum with respect to the structural asymmetry parameter (δ) of the metamaterial. The red lines and green line represent increasing and decreasing pathways for the asymmetry parameter, respectively.

Supplementary Figure 14.

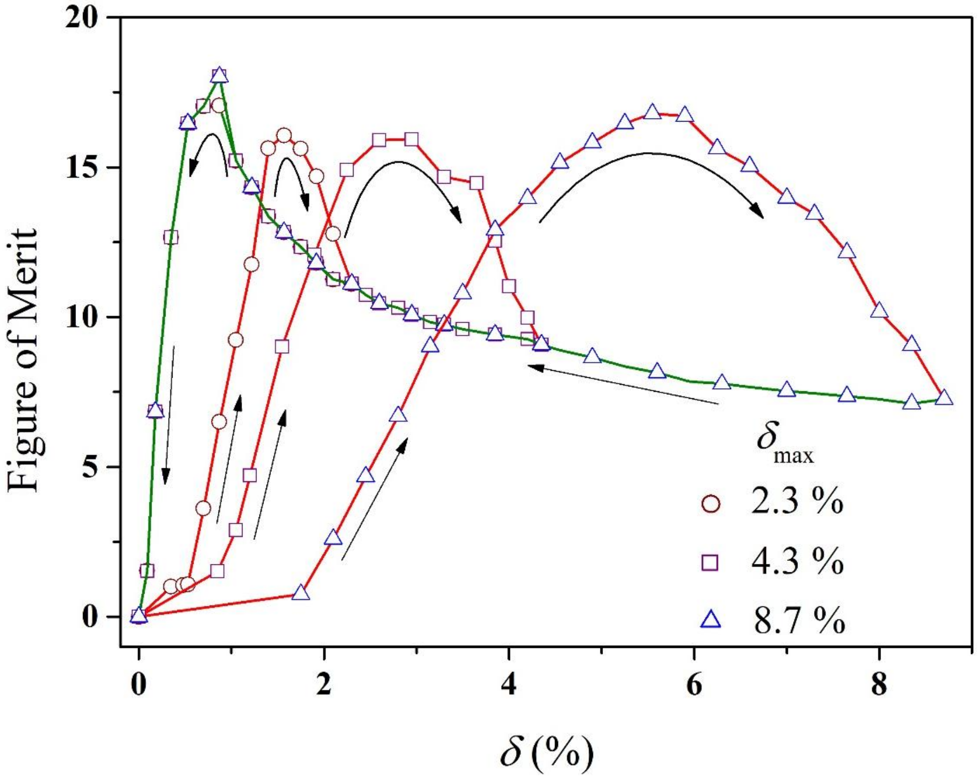

Tuneable MIO characteristics in FoM. Defined FoM calculated from the numerically simulated Fano resonance benefits in determining the favourable design parameters for achieving optimum devices for sensing and nonlinear applications. As shown in the figure, the observed multiple pathways for the increasing configuration of δ manifests tuneability of observed MIO curves along with the benefit of tuning the optimum asymmetry regime for realizing high performance devices for sensing and nonlinear applications, with tuneable mode volumes. The red lines and green line represent increasing and decreasing pathways for the asymmetry parameter, respectively.

Supplementary Figure 15.

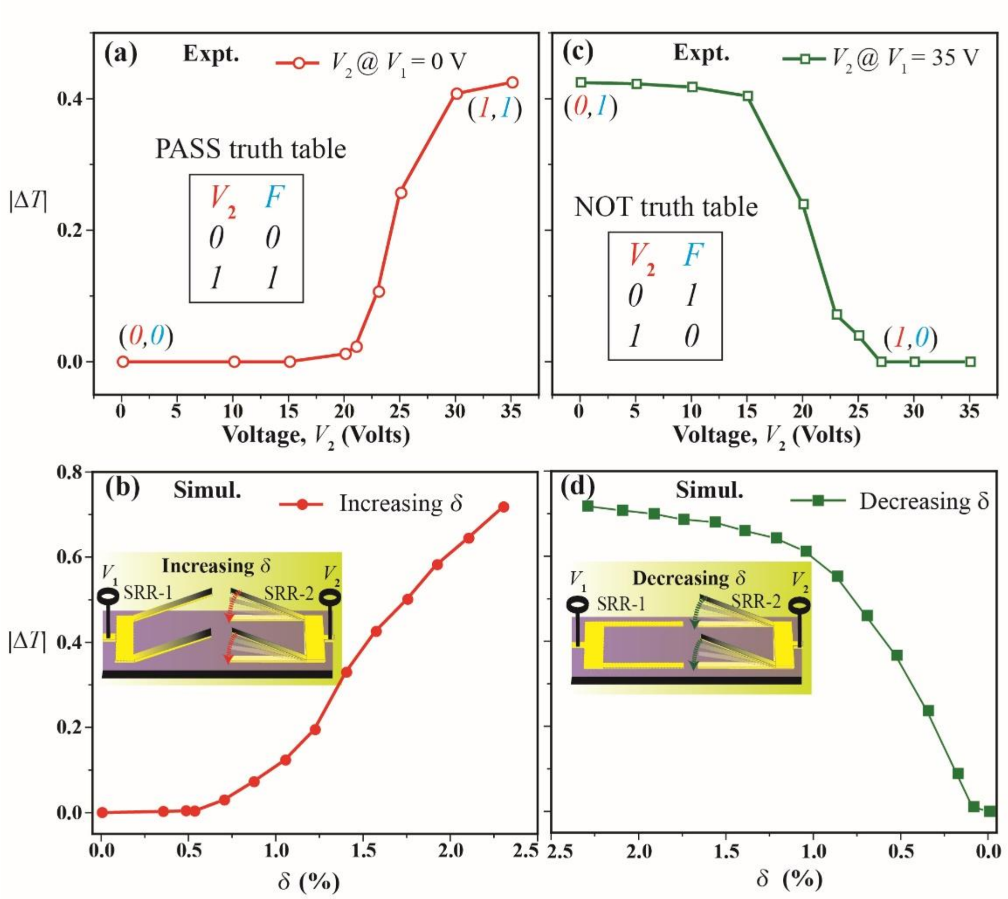

Single-input-output (SIO) states enacted PASS and NOT logic gates using the MEMS Fano-metamaterial. Here, we demonstrate the system exhibiting the XOR functionality that can also be used as the buffer/PASS logic operator by closing the input (V_1) having the low ('*0*') logical state (released state), while the other input (V_2) is varied, as shown in Figs. (a) and (b), where the operation does not alter the input state and output logic state is same as the input logic state. On the other hand, by closing the input possessing high ('*1*') logic state and other input being varied, one can realise NOT logic operation, as shown in (c) and (d). This condition leads to the inverter or the NOT gate possessing high contrast output intensity. Hence, based on the state of one of the SRRs (released or snapped), the control of other SRR (say, SRR-2) leads to a PASS or NOT logic operation, and hence this feature shows the pre-selection of operation between PASS to NOT gating logic through the added control signal (V_1).

Supplementary Figure 16.

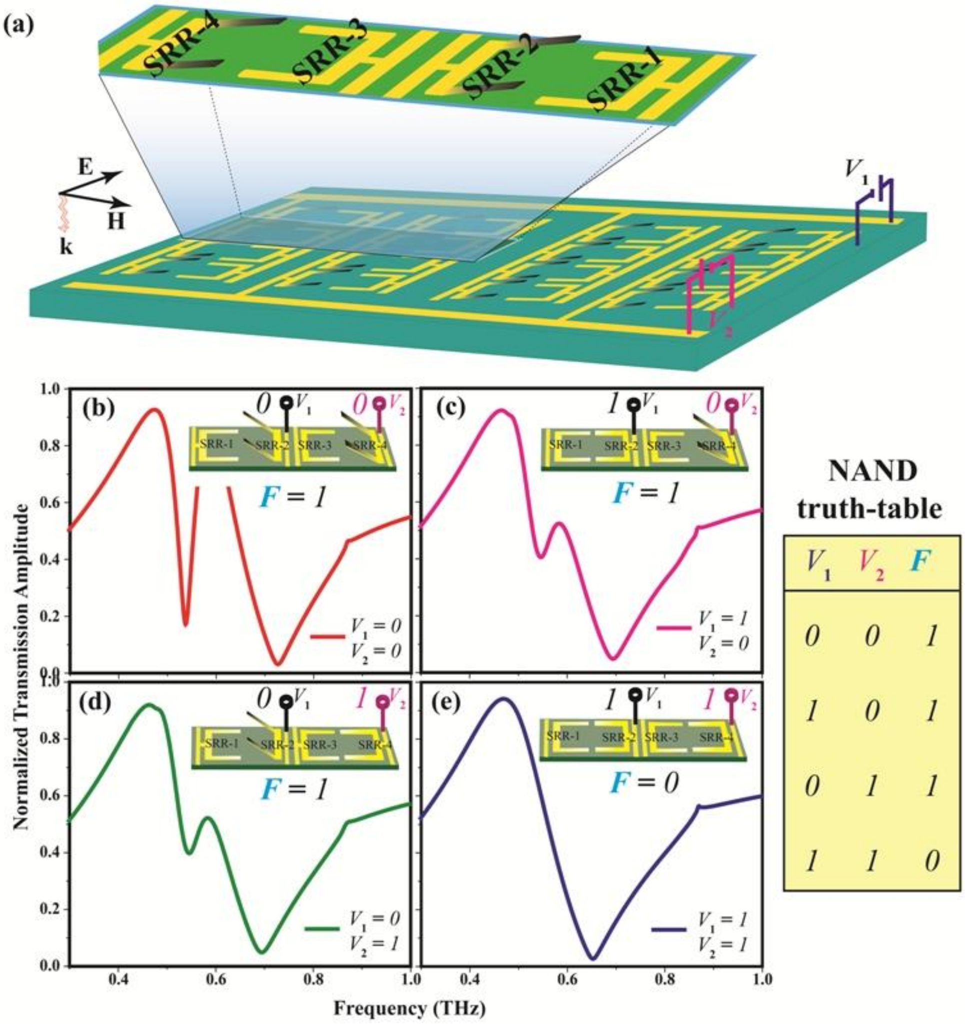

V_1	V_2	F
0	0	1
1	0	1
0	1	1
1	1	0

Far-field operation of the NAND gate using the MEMS Fano-metamaterial. Here, we conceptually show the NAND logical operations in the far-field intensity spectrum using the same geometrical design and concepts of MEMS Fano-metamaterial but by combining two unit-cells to make one composite unit-cell (super-cell) consisting of two Fano meta-molecules (a pair of SRR-1 - SRR-2 and SRR-3 - SRR-4), as shown in the Fig. (a). By independently controlling the SRRs present within the two Fano meta-molecules using the voltage sources (V_1 and V_2) the NAND logical function is realized by the THz readout pulse in the form of presence and absence of Fano resonance in the far-field intensity spectrum. As shown in the Fig. (a), for realizing NAND functionality, the initial configuration of the super-cell of the device is designed such that one of the two resonators in the Fano meta-molecule

(say, SRR-1 and SRR-3) are attached on the substrate and other two resonators (say, SRR-2 and SRR-4) are in the released states and are connected to voltage controls (V_1 and V_2) as two logical inputs. The geometrical dimensions of the metamolecule resonators in the super-cell are same as the MEMS Fano-metamaterial design discussed in the manuscript, while the periodicity of the structure along the *x*-axis is doubled (p_x = 220 μm & p_y = 75 μm) to form the super-cell consisting of a pair of Fano metamolecules. The far-field transmission spectra of the device are shown in Fig. (b-e), where the transmission spectrum depicted in (b) shows the strong excitation of Fano resonance feature at 0.53 THz observed in the far-field for no voltage inputs ($V_1 = 0$ & $V_2 = 0$), that results in the true output state of the device ($F = 1$). Similarly, for the device configurations with the voltage applied either on SRR-2 or on SRR-4 ($V_1 = 1$ and $V_2 = 0$ / $V_1 = 0$ and $V_2 = 1$), there is weak excitation of the Fano resonance at 0.53 THz, which signifies the true output state ($F = 1$). The reduced resonance strength in Fano resonance is caused due to the 50% reduction in the number of resonant Fano meta-molecules. For the last configuration, when both voltages ($V_1 = 1$ & $V_2 = 1$) are applied on the SRR-2 and SRR-4 of metamolecules, the Fano resonance features completely disappears, thereby resulting in the false output state ($F = 0$) of the optical readout. Thus, the proposed electro-optical feature of the MEMS Fano metamaterial forms the NAND logical operation, with possessing the true output states when either one/both input states are false.

Supplementary Figure 17.

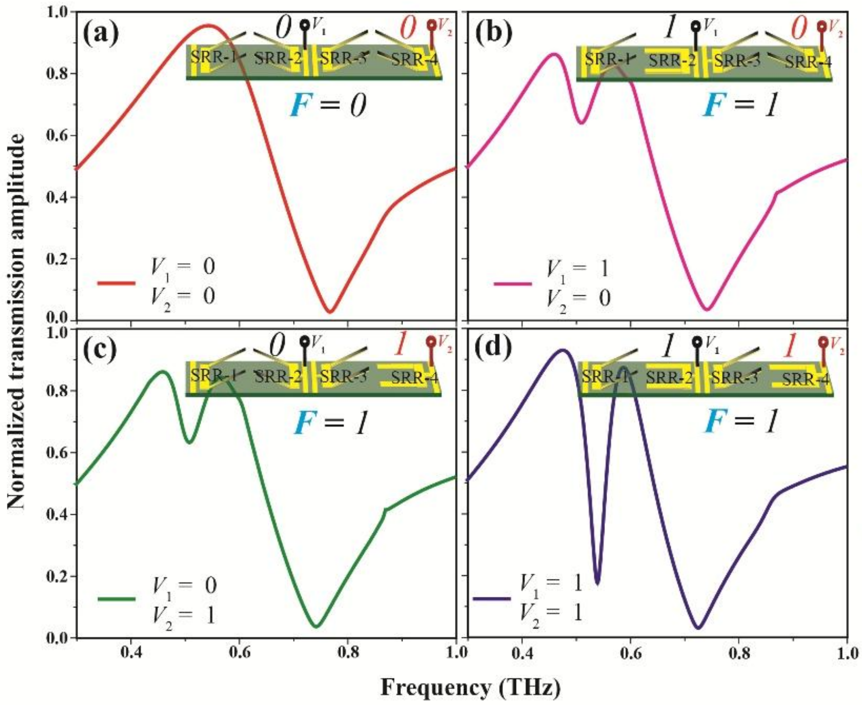

V_1	V_2	F
0	*0*	*0*
1	*0*	*1*
0	*1*	*1*
1	*1*	*1*

Far-field operation of the OR logic gate using the MEMS Fano-metamaterial. The figure represents the numerically simulated transmission spectrum of the metamaterial structures depicted in the inset of the respective plots. The electro-optical operation of the device results in the OR truth table for the input states of V_1V_2 = *00*, *10*, *01* and *11* giving the outputs as F = *0*,*1*,*1* and *1* respectively, in the form of true (F = *1*) and false (F = *0*) states of Fano resonance feature in the far-field amplitude spectrum. The OR logic operation is complementary to the NAND functionality of the proposed MEMS Fano-metamaterial, where, if the states of SRR-1 and SRR-3 are kept in the OFF state (released) instead of ON state (snapped down, in the case of NAND), the OR logic operation can be realized based on the control inputs to SRR-2 and SRR-4 and readout using the THz beam in the far-field amplitude spectrum.

Supplementary Figure 18.

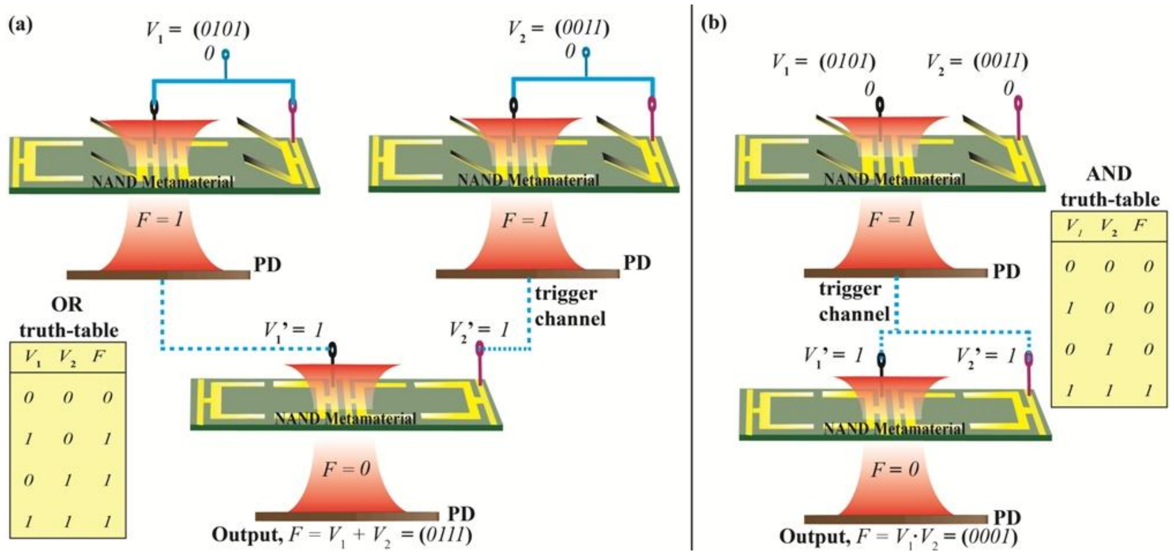

Cascading of metamaterial NAND logic gates in the far-field. Here, we provide a realistic schematic for achieving cascading of the far-field NAND metamaterial devices to form OR (Fig. (a)) and AND (Fig. (b)) logic outputs. The cascading of the gates would work similar to the electronic or optical counterparts. However, the proposed cascading of NAND metamaterial is unlike the conventional transistor or waveguide based cascading of gates, where either solely electrical or optical channels are used to realize the cascading networks. In the current device, the optical readout from the metamaterial is converted to electrical signals using the standard photodetectors and used as the trigger to the electrical inputs of the cascaded NAND metamaterial. Further, such cascading can assist in realizing other basic logic gates and complex sequencing that could merit the realization of digital sequences using metamaterials.

References:

1. W-H. Chut, M. Mehreganyt and R. L. Mullen. Analysis of tip deflection and force of a bimetallic cantilever microactuator, *J. Micromech. Microeng*. **3** 4-7 (1993).
2. L. Cong, M. Manjappa, N. Xu, I. Al-Naib, W. Zhang, and R. Singh. Fano Resonances in Terahertz Metasurfaces: A Figure of Merit Optimization. *Adv. Opt. Mater*. **3** (11), 1537–1543 (2015).
