# Supplementary material for: Reconfigurable MEMS Fano metasurfaces with multiple-input–output states for logic operations at terahertz frequencies
Source: Nat Commun. 2018 Oct 3;9:4056. doi: 10.1038/s41467-018-06360-5 (PMC6170453; doi:10.1038/s41467-018-06360-5)
Supplement: Supplementary file 3 — Description of Additional Supplementary Files [file 41467_2018_6360_MOESM3_ESM.pdf]

Description of Additional Supplementary Files

File Name: Supplementary Movie 1
Description: Non-Volatile operation of MEMS Fano device.

File Name: Supplementary Movie 2
Description: Volatile operation of MEMS Fano device.
